# Review on Development and Dental Applications of Polyetheretherketone-Based Biomaterials and Restorations

**DOI:** 10.3390/ma14020408

**Published:** 2021-01-15

**Authors:** Ludan Qin, Shuo Yao, Jiaxin Zhao, Chuanjian Zhou, Thomas W. Oates, Michael D. Weir, Junling Wu, Hockin H. K. Xu

**Affiliations:** 1 Department of Prosthodontics, School and Hospital of Stomatology, Cheeloo College of Medicine, Shandong University & Shandong Key Laboratory of Oral Tissue Regeneration & Shandong Engineering Laboratory for Dental Materials and Oral Tissue Regeneration, Jinan 250012, China; lucky_qinludan@mail.sdu.edu.cn (L.Q.); sure_1031@163.com (S.Y.); 2 Research Institute of Polymer Materials, School of Materials Science and Engineering, Shandong University, Jinan 250061, China; jiaxin_zhao@outlook.com (J.Z.); zhouchuanjian@sdu.edu.cn (C.Z.); 3 Department of Advanced Oral Sciences and Therapeutics, School of Dentistry, University of Maryland, Baltimore, MD 21021, USA; TOates@umaryland.edu (T.W.O.); michael.weir@umaryland.edu (M.D.W.); hxu@umaryland.edu (H.H.K.X.); 4 Center for Stem Cell Biology & Regenerative Medicine, University of Maryland School of Medicine, Baltimore, MD 21201, USA; 5 Marlene and Stewart Greenebaum Cancer Center, University of Maryland School of Medicine, Baltimore, MD 21201, USA

**Keywords:** polyetheretherketone, biomaterials, dental applications, surface modifications, adhesive properties

## Abstract

Polyetheretherketone (PEEK) is an important high-performance thermoplastic. Its excellent strength, stiffness, toughness, fatigue resistance, biocompatibility, chemical stability and radiolucency have made PEEK attractive in dental and orthopedic applications. However, PEEK has an inherently hydrophobic and chemically inert surface, which has restricted its widespread use in clinical applications, especially in bonding with dental resin composites. Cutting edge research on novel methods to improve PEEK applications in dentistry, including oral implant, prosthodontics and orthodontics, is reviewed in this article. In addition, this article also discusses innovative surface modifications of PEEK, which are a focus area of active investigations. Furthermore, this article also discusses the necessary future studies and clinical trials for the use of PEEK in the human oral environment to investigate its feasibility and long-term performance.

## 1. Introduction

**Figure 1:**
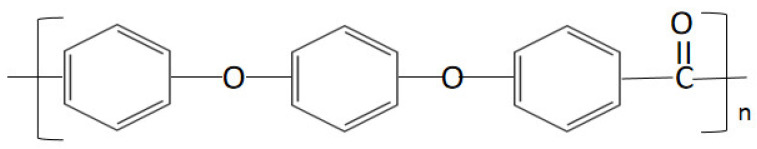
Chemical formula of polyetheretherketone (PEEK) Polyaromatic semi-crystalline thermoplastic polymer with chemical structure (–C6H4–O–C6H4–O–C6H4–CO–)n. (Reproduced from [1], with permission from © 2012 Elsevier).

Polyetheretherketone (PEEK) is an aromatic, semi-crystalline linear thermoplastic polymer [1] (chemical formula shown in Figure 1). As a member of the polyaryletherketone (PAEK) family, PEEK was developed from bisphenol salts and aromatic dihalides via nucleophilic substitution. With the participation of Williamson ether, the bisphenol salt was produced in situ from bisphenol with either added sodium or added alkali metal carbonate or hydroxide [2]. This chemical structure provides the material with stability at high temperatures (exceeding 300 °C), resistance to chemical and radical damage, greater strength (on a per mass basis) and compatibility with reinforcing agents such as glass and carbon fibers [2,3,4,5].

PEEK has a wide range of applications, including automotive, electronics, aircraft and turbine blades. In the medical field, PEEK has been used in orthopedic treatments, cardiac operations, maxillo-facial surgeries, spinal operations and cranioplasty [6]. PEEK is an essential high-performance dental material, with applications in oral implant [7,8,9], prosthodontics [10,11,12,13,14] and orthodontics [15,16,17]. Vitro and vivo studies demonstrated a satisfactory biocompatibility of PEEK [18,19,20,21]. Moreover, compared with current metal alloys, the compatibility of the elastic modulus between the PEEK biomaterials and human bone may lessen the stress shielding effects on the surrounding bone [4,22,23,24,25].

PEEK has an opaque or greyish color [26] and is not suitable for aesthetic restorations on anterior teeth [27,28]. Therefore, approaches of bonding with resin composites or veneering have been used to obtain aesthetic results [29,30,31,32]. However, these methods also face additional challenges. Achieving satisfactory bond strength of PEEK to resin composites remains difficult because of the low surface energy and inertness of PEEK [30,32,33]. Studies have been conducted to overcome these shortcomings of PEEK [13,34,35,36]. This article reviews the most recent developments of PEEK-based biomaterials in dental applications, novel improvements in properties and innovative engineering methods for surface modifications.

## 2. Materials and Methods

For this review, only studies published in English were included, and the search was performed in December 2020. PubMed/MEDLINE and Web of Science databases were used. The following keywords were used in various combinations: “PEEK, Polyetheretherketone, PEEK composites, properties, applications, dentistry, oral application, polymeric material, implantology, orthodontics, prosthodontics, adhesive properties, adhesion, bonding strength, surface modification, laser treatment, plasma treatment, abrasion treatment, acid etching”. We divided the papers into several groups for: properties, applications including orthodontics, implantology, prosthodontics and adhesion of PEEK to dental composites.

## 3. Properties of PEEK-Related Biomaterials

Due to its excellent mechanical properties, PEEK has been used as an implant material, computer-aided design and computer-aided manufacture (CAD/CAM) material, coating material and abutment material [37]. PEEK has a similar Young’s modulus (3–4 GPa) to human bone [23,26,38,39]. Its capability to be combined with other materials was a favorable advantage of this material [40]. For example, PEEK composites reinforced with carbon fibers (CFR-PEEK) had a higher elastic modulus (18 GPa) [41], which is equivalent to human cortical bone and dentin [42]. CFR-PEEK exhibited less stress-shielding when compared to titanium implant [38], as shown in Table 1. Despite its low elastic modulus, the wear resistance of PEEK was comparable to that of metal alloys [43]. Furthermore, since it had good fatigue resistance and exhibited a low creep rate, PEEK was a popular bearing material [17,44]. It was reported that among three types of tested orthodontic wires, the highest flexural strength and creep resistance were observed with PEEK [17]. The tensile properties of PEEK matched those of enamel and dentin, which made it a potential alternative for the framework of prosthodontic restorations [38,43].

The chemical resistance of PEEK helped minimize its biocorrosion, thus avoiding the release of toxic by-products [45]. PEEK was not damaged by exposure to conventional solvents. In addition, due to its distinctive aromatic chemical structure, PEEK also exhibited excellent resistance to gamma and electron beams [46] which were used to sterilize medical devices. Furthermore, due to its radiolucency, PEEK created no artifacts on the magnetic resonance imaging and X-ray radiographs [47]. PEEK had a low water-solubility of 0.5%, with long-term water exposure causing no chemical damage, even at temperatures of up to 260 °C [18]. However, PEEK was bioinert and hydrophobic, thus limiting its direct bone contact ability [48,49]. The improvement of the bioactivity of PEEK implants is much needed and requires further research [50].

PEEK is metal-free and aesthetic and has found use in prosthodontic applications including fixed or removable prostheses [12,15,51]. To acquire a more aesthetic effect, resin composites have been used to cover the opaque and gray PEEK [32]. In addition, surface modifications of PEEK have been investigated to achieve a strong adhesion between PEEK and resin composites.

Furthermore, the satisfactory and excellent biocompatibility of PEEK has been reported [52]. In vitro study showed no evidence of mutagenic or cytotoxic activity of PEEK on human organisms [21,53]. Excellent performance was also observed in investigations on the in vitro cellular biocompatibility for CFR-PEEK [54]. In addition, according to the bone and soft biocompatibility of in vivo studies, there was no toxic damage on fibroblasts or osteoblasts caused by PEEK [19,55].

## 4. Applications of PEEK in Orthodontics

Due to the increasing aesthetic demand of patients, non-metallic orthodontic wires are becoming increasingly popular [56]. PEEK is highly promising as an excellent alternative to metal alloys. For dental applications, PEEK overcomes some of the main shortcomings of metal alloys, such as poor aesthetics, metal allergies, releasing metal ions and corrosion in the oral environment [15,16,57]. PEEK also avoids the interference of metals with magnetic resonance imaging [17].

Tada et al. [15] evaluated the load-deflection features and frictional properties of PEEK wires, concluding that PEEK wires were suitable as orthodontic appliances. The load-deflection curves displayed similarities among the tested wires, while the differences in the permanent deformation and static friction of all the materials were insignificant. Maekawa et al. [17] studied the three super engineering plastics (SEPs): PEEK, polyetheretherketone (PES) and polyvinylidene fluoride (PVDF), as the base material of orthodontic wires. Compared with other materials, PEEK has good creep resistance, the highest bending strength and lower water absorption, which make it a suitable substitute for metal-free orthodontic wire.

**Figure 2:**
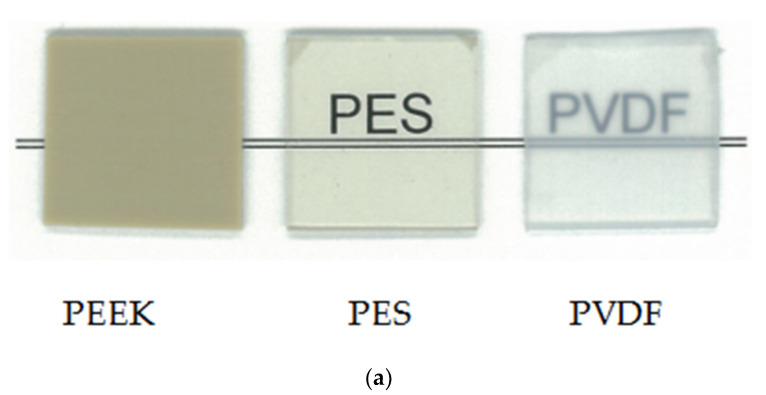

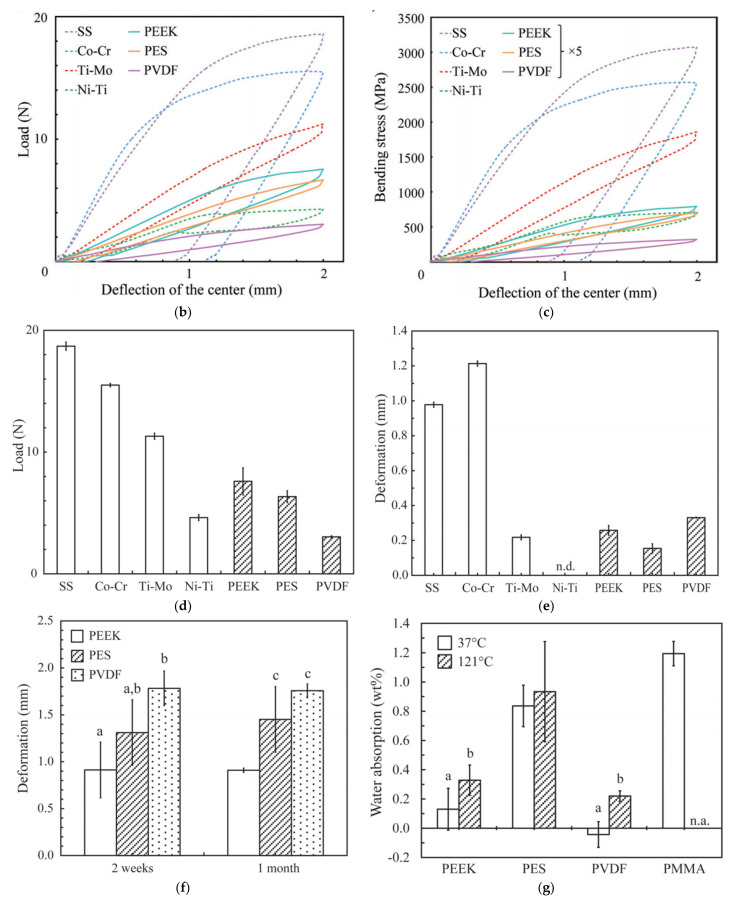
Mechanical properties of PEEK orthodontic wires. (**a**) Color properties of SEPs (1.0 mm thick). Curves of load-deflection (**b**) and bending stress-deflection (**c**) for the specimens via the three-point bending test. (**d**) Bending load at 2.0 mm deflection and (**e**) permanent deformation after three-point bending test. Data are mean ± SD from five independent replicates. Tukey-Kramer HSD test indicated significant differences between each pair (*p* < 0.05). ‘n.d.’ means ‘not detected’. (**f**) Permanent deformation after 2.0 mm bending creep test (for 2 weeks and 1 month). (**g**) Water absorption at 37 and 121 °C (autoclaving) for 10 days. Data are mean ± SD from four independent replicates. ‘n.a.’ means ‘not available’. Bars annotated with the same letter are not significantly different, as assessed by Tukey-Kramer HSD test (*p* > 0.05). (Reproduced from [17], with permission from © 2015 Elsevier).

Figure 2a shows the color outward appearance of three SEPs (1.0 mm thick). Better aesthetic appearance was observed in PES and PVDF, rather than the grey and opaque PEEK specimens. As proven in Figure 2b, the typical load–deflection curves of the examined wires indicated that stainless steel (SS) and cobalt-chromium (Co-Cr) wires exhibited greater flexural loads and larger everlasting deformations than the rest of material. In term of deformation, the Nickel-titanium (Ni-Ti) wire had a typical load-deflection curve with no permanent deformation, while SEP wires had the opposite results. Figure 2c displays the bending stress-deflection curves of the specimens. Bending load at 2.0 mm deflection in the three-point test is shown in Figure 2d. PEEK had the greatest flexural load in all of the SEPs. Figure 2e indicates the permanent deformation after the 2.0 mm bending load. Perpetual deformation has not been found in Ni-Ti specimens. On the contrary, less deformation has been observed in SEP samples (PEEK, 0.2 mm; PES, 0.1 mm; PVDF, 0.3 mm). Figure 2f indicates creep deformation of SEP wires after the bending creep test. Among the SEPs, PEEK wires had less creep deformation (less than 1.0 mm deformation after 1 month of bending at 37 °C).

Water absorption at 37 and 121 °C (autoclaving) for 10 days is displayed in Figure 2g. PEEK exhibited a much smaller water absorption (no more than 0.2 wt.% at 37 °C and 0.4 wt.% at 121 °C). Minimal water absorption at 37 °C was found in PVDF due to its hydrophobic nature. In addition, a recent study reported that orthodontic archwires with the PEEK tube had the right combination of aesthetics and functional properties in orthodontic appliances. Passing the archwire through the PEEK tube also reduced the frictional force between the bracket and wires [16]. In addition, PEEK could be used as coatings on Ni-Ti alloy wires to enhance the corrosion resistance and improve the mechanical properties [58]. However, the long-term oral exposure might cause severe deterioration to the stability of PEEK coating [59]. Therefore, further studies are required to evaluate the long-term stability of PEEK-coated orthodontic wires.

Therefore, PEEK makes the treatment more acceptable for patients with aesthetic availability and thus could be regarded as feasible orthodontic wires [17,38]. However, PEEK wire has not been widely used clinically, and there are insufficient data on its long-term mechanical and aesthetic properties in the human oral environment. Moreover, the load-deflection characteristics and static friction of PEEK orthodontic wires might be influenced by oral activities, such as eating, brushing or removing food colorants and stains. As a result, further investigations are required to take the simulated clinical conditions including wet conditions into account [15]. For PEEK orthodontic wires, long-term investigations of the mechanical properties under clinical loading conditions are needed as well.

## 5. PEEK Materials in Oral Implantology

PEEK implant has emerged as a substitute for patients with bruxism or allergic reactions to metals [60]. In addition, the outstanding mechanical properties, superior biocompatibility and stiff semi-crystalline nature with bone-like hardness have made PEEK a promising biomaterial for orthopedic implant applications [23,61].

### 5.1. PEEK as Dental Implants

PEEK could be recommended for applications in oral implantology. However, dental implants fabricated by PEEK have not been extensively used clinically [38], compared with orthopedic implants [62,63,64]. Stress-shielding is a mechanical phenomenon that could be interpreted as the adjustment in mechanical stimulus in the bone surrounding to the implant [65]. Stress shielding considerations indicated that the compliance of dental implant material is required to match the host bone [66]. Otherwise, in case of stresses exceeding the level of the bone strain, the bone around an implant would suffer possible complications such as per-implant bone resorption or reduction [67]. Stresses below the level would exert favorable outcomes for the implant and stimulate bone remodeling phenomena [9,66]. The stiffness of the dental implants would be decreased and then the bone apposition of surrounding bone could be observed based on finite element analysis (FEA) [8]. A study also demonstrated the same result that the low stress shielding effects of PEEK material was beneficial to prolong the implant lifespan [68]. As a result, a more well-proportioned stress distribution of PEEK and its composites to the adjacent bone requires further research.

Furthermore, by adding carbon fibers, the elastic modulus of PEEK could be reinforced to up to 18 GPa [41]. Adjusting the fiber length and orientation would make it better suited to the cortical bone or titanium alloy [47]. A study demonstrated that stress distribution in CFR-PEEK dental implant had higher stress peaks due to a reduced stiffness compared to titanium. This type of implant presented inhomogeneous stress distribution in the cervical area and cortical bone than the titanium implant [41]. Furthermore, similar results were observed in another study. Compared with the CFR-PEEK implant, titanium exhibited a more homogenous stress distribution due to its smaller deformation, as reported by Sarot et al. [69].

**Figure 3:**
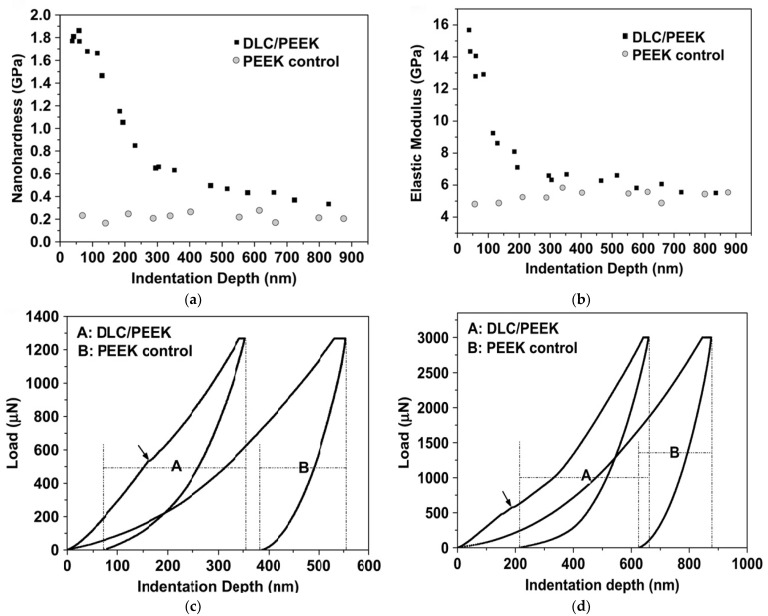
Nanohardness, elastic modulus and load-displacement curves of DLC/PEEK and PEEK control. (**a**) Nanohardness and (**b**) elastic modulus of DLC/PEEK and PEEK control as a function of indentation depth. Comparison of load-displacement curves of DLC/PEEK and PEEK control at the peak indentation load at (**c**) 1300 mN and (**d**) 3000 mN. (Black arrows: ring-like through-thickness cracking) (Reproduced from [22], with permission from © 2010 Elsevier).

The diamond-like carbon (DLC) exhibited a more identical elastic modulus to the cortical bone than PEEK as suggested by Wang et al. [22]. They synthesized DLC/PEEK to enhance the stability and surface properties of PEEK in bone implant. PEEK was coated with DLC by plasma immersion ion implantation and deposition (PIII&D). The system had comparable elasticity to cortical bone which could prevent the resorption of adjacent bones due to the reduced stress shielding. Figure 3a,b, respectively, indicate the nanohardness and elastic modulus values of the DLC/PEEK and PEEK control along with a function of the indentation depths from 50 to 900 nm. After coating with DLC, the surface hardness enhanced from 0.2 to 1.9 Gpa, and the surface elastic modulus increased from 5 to 16 GPa.

Figure 3c,d exhibit the load-displacement curves acquired at loadings of 1300 and 3000 mN. Under the same load, a smaller indentation depth and larger elastic recovery were observed in the DLC/PEEK specimens.

**Figure 4:**
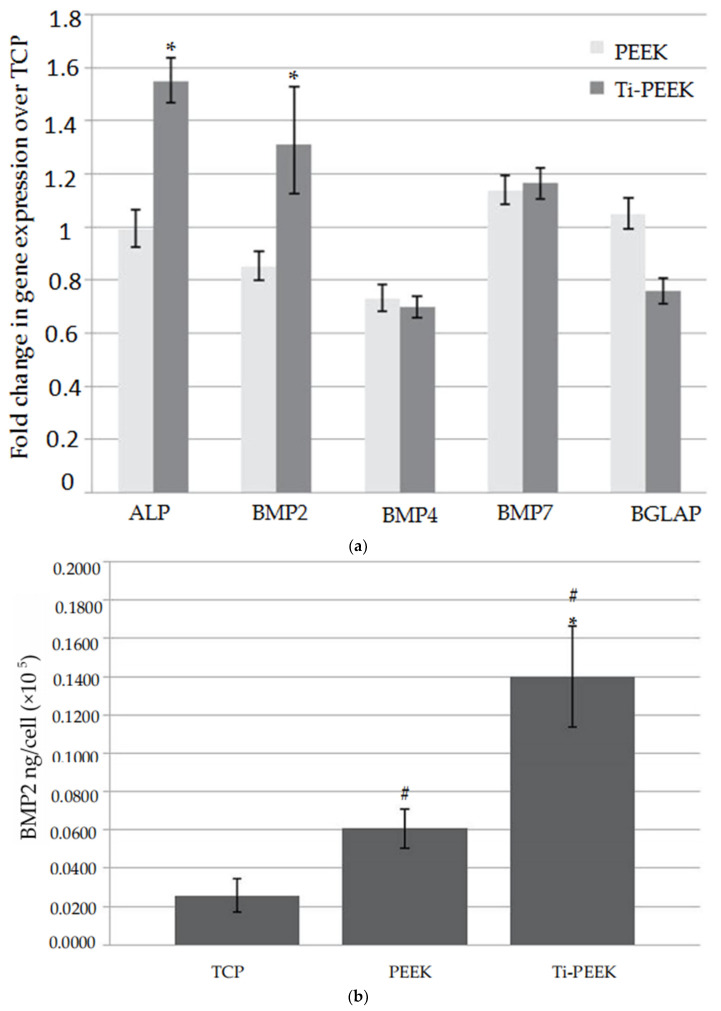
Gene expression of bone markers; protein levels of Ti-PEEK and PEEK groups. (**a**) Fold changes in gene expression of bone markers (normalized to GAPDH) over TCP control (calibrator = 1) in osteoblast-like cells cultured on Ti-PEEK and PEEK surfaces; * 95% CIs of mean fold change values of Ti-PEEK and PEEK do not overlap; (**b**) BMP-2 protein levels secreted by osteoblast-like cells cultured on Ti-PEEK, PEEK and TCP surfaces; * *p* < 0.05 vs. PEEK; # *p* < 0.05 vs. TCP. (Reproduced from [72], with permission from © 2018 Dovepress). GAPDH: glyceraldehyde 3-phosphate dehydrogenase.

**Figure 5:**
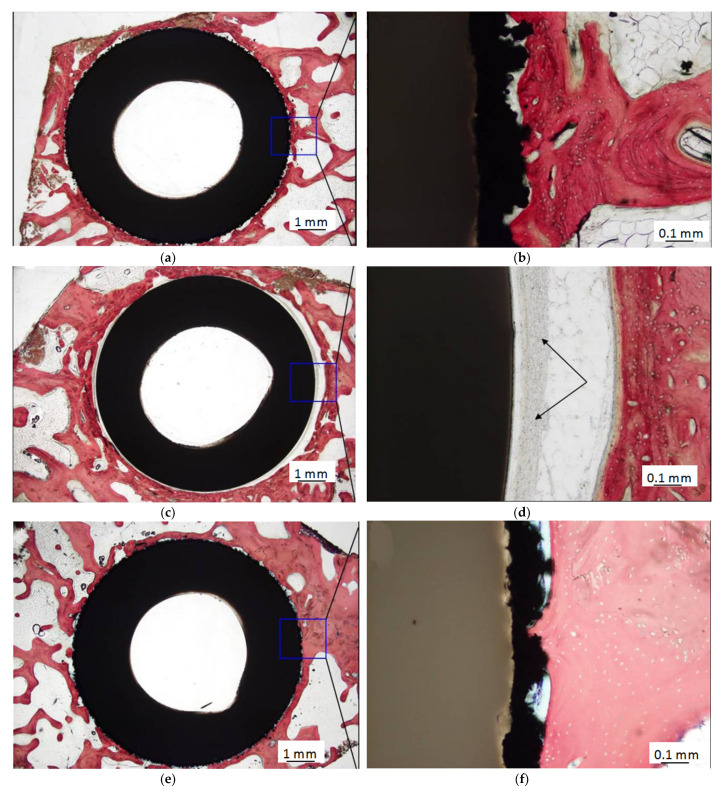

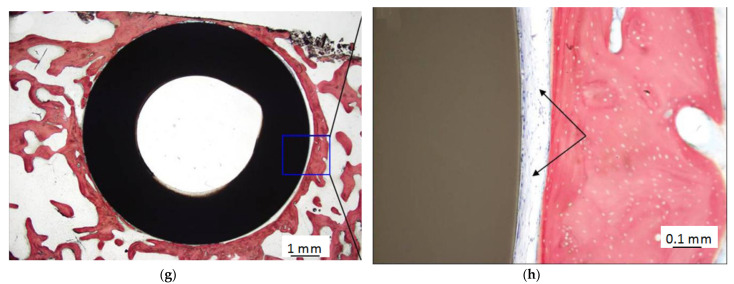
Histology results for Ti-PEEK implants and uncoated PEEK implants. 12 weeks for Ti-PEEK implants (**a**,**b**) and uncoated PEEK implants (**c**,**d**); 24 weeks for Ti-PEEK implants (**e**,**f**) and uncoated PEEK implants (**g**,**h**). Black arrows: fibrous connective tissue. H&E stain. Bar = 1 mm (**a**,**c**,**e**,**g**); 0.1 mm (**b**,**d**,**f**,**h**). (Reproduced from [72], with permission from © 2018 Dovepress).

Additionally, when compared to titanium, PEEK implants had minimal osteoblast differentiation [70], and they did not display any osteoconductive properties [49,71]. However, favorable bony deposit could be found in the titanium-coated PEEK (Ti-PEEK), as shown by Cheng et al. [72]. Gene expression of bone formation markers, protein levels and histological images of the Ti-PEEK and PEEK groups are shown in Figure 4 and Figure 5. In Figure 4a,b, the markers were expressed on the surfaces of samples at different degrees when compared to the tissue culture plastic (TCP) control. The expression of alkaline phosphatase (ALP) and bone morphogenetic protein-2 (BMP-2) genes, activity and BMP-2 protein levels was higher on Ti-PEEK. In Figure 5, the histology results show that bony apposition adjacent to the Ti-PEEK surface was observed at the 12 and 24 week time points, while the PEEK group exhibited less bony apposition.

Sulfonation treatment [45,73,74,75], plasma immersion ion implantation [39,76] and bioactive coatings [72,77,78] are commonly used in surface modification of PEEK implants. In addition, by blending with fine filler particles to synthesize PEEK composites [25,79,80,81,82], bioactive implants with good osseointegration could be produced.

**Figure 6:**
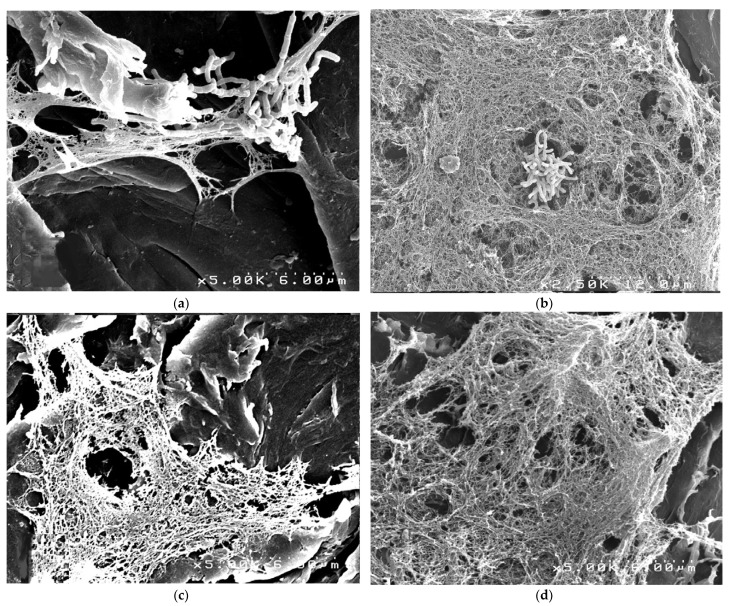
Representative Scanning Electron Microscopy (SEM) biofilm images on PEEK. (**a**) 8 h, (**b**) 12 h, (**c**) 16 h and (**d**) 20 h of adhesion. Biofilm was observed to form on PEEK only. (Reproduced from [84], with permission from © 2020 Elsevier).

PEEK dental implants tend to have oral biofilm formation in vivo [5,48], which increases the risk of bacterial infection around the implants [46]. Several strategies could be adopted to solve this problem. These include PEEK sulfonation treatment, coatings, incorporation of bioactive composites in PEEK substrate or on the surface and introduction of reinforcement agents to produce nanosized composites [83]. Furthermore, Garcia et al. [84] reported that, compared with metallic implants, PEEK implants could boost early intraoperative bacterial colonization and subsequent infection. *C. acnes* was found to form biofilms on PEEK as observed by Scanning Electron Microscopy (SEM) (Figure 6). In Figure 6a, biofilm formation on PEEK was exhibited at 8 h following inoculation to PEEK. As displayed in Figure 6b–d, an increasing adherent bacterial concentration from 12 to 20 h of adhesion occurred. Furthermore, instead of anchoring to the surrounding bone, PEEK and CFR-PEEK implants tend to become encapsulated by fibrous tissue and/or colonized by bacteria because of foreign body reaction [46]. To enhance the antibacterial activity, a polyetheretherketone/nano-fluorohydroxyapatite (PEEK/nano-FHA) biocomposite with the ability to prevent bacterial proliferation and biofilm formation was developed by Wang et al. [48].

### 5.2. PEEK as Dental Implant Abutments

Metals such as titanium and ceramics such as zirconium oxide can be used for dental implant abutments. Recently, PEEK was introduced as a restorative material in implant dentistry and increasingly employed as implant abutment material [85,86]. Hahnel et al. [85] analyzed the biofilm formation in implant abutment materials of titanium, zirconia and PEEK. PEEK featured favorable properties as an abutment material, having lower biofilm formation on its surface than the traditional abutment materials of titanium and zirconia. The biomechanical behaviors of resin-matrix ceramics and PEEK customized abutments in terms of stress distribution in implants and peripheral bone were evaluated by Kaleli et al. [37]. Using the PEEK-customized abutment increased the stress concentration when restored with crowns. In addition, the material of the healing abutment (PEEK or titanium) did not significantly influence the soft and hard tissue during the healing period of 3 months, as shown by Koutouzis et al. [87].

PEEK reinforced with titanium abutments serves as an effective alternative to traditional abutments, given its excellent biocompatibility, and could preserve the bone height and stabilize the soft tissue [88]. According to the fracture strength of implant-supported resin crowns, no significant difference was observed between PEEK abutments and titanium temporary abutments, except for central incisors, as reported by Santing et al. [86].

Currently, due to the accessibility and high accuracy, intraoral digital impression systems have been introduced into the fabrication of dental implant restorations [89,90]. Implant scanbody (ISB) manufactured with PEEK material is conducive to enhancing the accuracy of digital impression [91]. The highest accuracy of the impression on both linear and angular measurements was observed in PEEK material, followed by titanium and PEEK-titanium. As a result, the ISB materials would potentially display various effects on the accuracy of impression. The optical properties of PEEK could enhance the intraoral digital optical scanning to acquire more points from the ISB surface, leading to a more accurate scanning result. The fit and wear-resistant properties of PEEK material may influence the process of screwing onto the implant [91].

In summary, there are only a few studies on the clinical evaluation of PEEK abutments, and the longest research only lasted for several months [47,87]. More clinical trials are vital to evaluate the hard and soft tissue responses to PEEK materials and their lower biofilm formation.

### 5.3. PEEK as Abutment Crown and Abutment Screw

To date, PEEK has been used for the manufacturing of provisional implant crowns. The screw-retained implant crown fabricated by PEEK has potential in implant-supported restorations [26]. In addition, as an important factor, fracture strength deserves to be considered to evaluate the clinical service and failure rates of prosthetic materials. Implant-supported three-unit fixed partial denture (FPD) from PEEK could bear high occlusal forces such as excessive crown height space (CHS) [92]. Furthermore, due to the high elastic modulus of the metal framework and the mobility of the abutment teeth, resin-bonded restorations are prone to unfavorable stress concentrations at the bonded interface; therefore, the risk of debonding could be increased. Hence, Zoidis et al. [12] fabricated an interim 3-pontic resin-bonded FPD using a PEEK framework veneered with resin composites after implant placement. It shows superiority over metal ceramics or ceramics in dampening the occlusal forces and reducing debonding rates. New PEEK-based materials reinforced with ceramics emerged with stronger mechanical properties as permanent restorations. Ceramic-reinforced PEEK crowns over titanium and zirconia abutments demonstrated high fracture resistance, which was equivalent to the commonly employed ceramic material, as shown by Elsayed et al. [93].

Abutment screw fractures are related to many factors, including the types of implant abutment screw materials. Using an assembly of an external hexagonal implant/UCLA-type abutment, Neumann et al. [94] compared the fracture resistance of implant abutment retention screws manufactured of titanium, PEEK and 30% carbon fiber-reinforced PEEK in vitro. All abutment screws manufactured from the above materials had fractures at the neck. Nonetheless, PEEK and 30% CFR-PEEK abutment screws exhibited lower fracture resistance than titanium. Stimmelmayr et al. [95] measured the abutment rotation and fracture load of two-piece zirconia implants screwed with gold, titanium and PEEK, respectively. The results showed no significant differences in the three tested materials. However, the group with PEEK screws showed lower fracture values when compared with the gold and titanium groups. The feasibility for PEEK as an abutment screw remains questionable and requires further testing.

Although PEEK attracts increasing clinical attention and interest, its poor integration into surrounding bone tissues caused by its biological inertness remains a challenge. This is especially true for the implant applications of PEEK in bacteria-infected operative regions. Further studies are required to enhance the infection resistance and osseointegration of PEEK [48]. Evidence of satisfactory osseointegration in preclinical practice and standard outcome measures is also required to ensure the use of PEEK implants as an alternative implant for clinical cases [38].

## 6. Effects of PEEK for Prosthodontics Applications

PEEK is increasingly used in the manufacture of removable and fixed prostheses [96], such as dental crowns, bridges and denture clamps in removable dental prostheses [38,97,98,99,100]. This is due to its advantages, including low potential to deduce an allergy, low water solubility, superior biocompatibility, high thermal and chemical resistance, moderate biofilm formation and excellent mechanical properties [97,98].

### 6.1. PEEK as a Removable Prosthesis Material

Currently, patients with missing teeth tend to choose implant restorations [101]. The success of implant dentistry has prompted the scope of aesthetic fixed prosthesis. However, there are still many patients who, for healthy, anatomic, psychological or financial reasons, are not suitable candidates for implants [14,102]. Those patients prefer to choose the removable partial denture (RPD), which could provide a conservative and cheap approach for replacing the lost teeth [101].

CAD/CAM technology has been used in prosthodontics [102,103]. Harb et al. [104] gave a clinical report on the fabrication of the PEEK framework of Kennedy Class I RPD by CAD/CAM milling technology. They suggested that milled PEEK might be a useful alternative framework material for RPD when restoring Kennedy Class I edentulous patients. Additionally, using CAD/CAM or traditional lost wax technique, the RPD framework manufactured by PEEK could be a substitute for the cobalt-chromium framework for patients who were allergic to traditional materials or more sensible in taste [11]. These cases indicated that this elastic material was beneficial to the periodontal health of abutment teeth, since it might decrease the distal torque and strain for the abutment teeth [105,106]. Similar results were found in another study [107]. However, a clinical study demonstrated that there were similar effects on oral health-related quality of life, patient satisfaction and periodontal outcomes for the PEEK denture frameworks, compared with cobalt-chromium denture frameworks [108].

In addition, Tannous et al. [14] suggested that the clasps of RPD fabricated by PEEK exhibited lower retention than the cobalt-chromium clasps. There was a need to evaluate the retention effects of PEEK clasps in clinical use. PEEK was unfit for the dental clasps since the maximum stress occurring during removal with higher undercuts was higher than the material strength [109].

The stress peaks and undercut for selected clasp materials were summarized in Table 2. It could be observed that the polyamide clasp with the lowest stress (17.1 MPa) caused less stress in the enamel surface (1.4 MPa) and the lowest reactional force (3.13 N) with an undercut of 0.25. The cobalt-chromium clasp with the highest stress (297.9 MPa) exhibited the highest stress in the enamel surface (46.4 MPa) and the highest reactional force (65.37 N) with a 0.75 undercut.

In contrast, Peng et al. [110] claimed that the superior flexibility and lower elastic modulus of PEEK had a deeper undercut than the cobalt-chromium alloy and could exert lower stresses on the abutments. Furthermore, according to the aesthetic field, clasps and occlusal rests fabricated by PEEK provided metal-free, satisfactory and aesthetic effects for patients with high aesthetic requirements [11,104].

### 6.2. PEEK Crowns

Crown restorations made from PEEK exhibit poor aesthetics when restoring anterior teeth. The greyish and opaque color has limited PEEK for crown restorations [29,31,96,111]. The PEEK framework combined with veneering could provide a satisfactory aesthetic outcome [12]. Double crown systems, including telescopic crowns with a 0° taper and conus crowns, are capable of providing retention for RPD due to their guidance, support and protection from dislodging movements [98]. In the double crown system, CAD/CAM manufactured PEEK secondary crowns exhibited sufficient and stable retentive force values even after artificial aging, which were equivalent to 10 years of clinical usage [97]. Another study by Merk et al. [99] similarly claimed that PEEK might be suitable for telescopic crown technique when being used on zirconia crowns. Stock et al. [98] also suggested that in combination with cobalt-chromium or zirconia secondary crowns, PEEK could be regarded as a suitable material of primary crown for RPD.

PEEK has similar wear resistance to metal alloys. However, the abrasion on natural teeth caused by PEEK crowns has not been investigated, especially when compared with conventional crown materials. As a result, evidence is required to prove the functional harmony between PEEK crowns and dentin and enamel [38].

### 6.3. PEEK as a Fixed Partial Denture Material

Both conventional fabrication process and CAD/CAM methods could be used to manufacture the fixed partial denture (FPD). The restorations could be more rapidly chair-side-fabricated via the CAD/CAM technique than the conventional process [38,112]. In addition, the prosthesis fabricated by the traditional procedure had a lower load-bearing capacity than that manufactured by CAD/CAM [113,114]. Stawarczyk et al. [100] showed that three-unit-reinforced PEEK FDP via CAD/CAM had a higher fracture resistance, stability and reliability than the pressed granular or pellet shaped restorations. In addition, load-bearing test results of PEEK (995.52 ± 78.1 N) indicated that it could be used as a substitute to the resin-based FPD material, as shown by Cekic-Nagas et al. [113]. Unfilled PEEK was used as a fixed partial denture framework and was reported in a clinical case. The framework was found to have satisfactory esthetic appearance, marginal fit, stability and light weight in the patient’s mouth [115].

## 7. Adhesion of PEEK to Dental Composites

PEEK has difficulty in bonding with resin composites due to its inert performance, low surface energy and surface modification-resistance [116]. Attention has been paid to enhance the adhesive properties of PEEK. Furthermore, the additional adhesive system was another issue that influenced the bonding strength of PEEK to the composites [31,34,117].

### 7.1. Airborne-Particle Abrasion

Airborne-particle abrasion could improve the micro-roughness and bonding surface area, and simultaneously clean the surface [116], thus enhancing the bond strength between veneering resin to PEEK [30]. Stawarczyk et al. [29] evaluated the influence of varying the pressure and particle grain sizes on the tensile bond strength (TBS) between PEEK and the veneering material. The pressure of air-abrasion had a corresponding influence on the bonding characteristics instead of particle grain sizes. The shear bond strength of this material was 10.81 ± 3.06 MPa via airborne-particle abrasion, and it still could be regarded as a feasible surface treatment. This was because materials with above 10 MPa shear bond strength were acceptable [118]. Other studies also suggested that airborne-particle abrasion had the ability to improve the bond strength of veneering resin to PEEK [30,119].

In addition to single treatment of airborne-particle abrasion, when combined with chemical acid etching, the adhesion between PEEK and dental resin composites could be achieved [1]. For example, Keul et al. [31] investigated the effects of air abrasion and air abrasion + etching with piranha solution on the enhancement of adhesion between PEEK and resin composites, and they found that the bonding strength was improved. The same method was also conducted by Hallmann et al. [1]. They abraded the surface of PEEK and used piranha solution to etch its surface in combination with Heliobond-like adhesive. This resulted in the highest bond strength of 21.4 MPa.

Further studies focusing on the combination of air abrasion and chemical treatment, such as acid etching, are required. After chemical treatment, more functional groups would appear if there is enough contact surface, which has a positive effect on the cross linking of polymeric materials. Moreover, the mechanical anchoring effects of the adhesive could be improved in the condition of a special surface structure derived from the air abrasion [1].

### 7.2. Plasma Treatment

**Figure 7:**
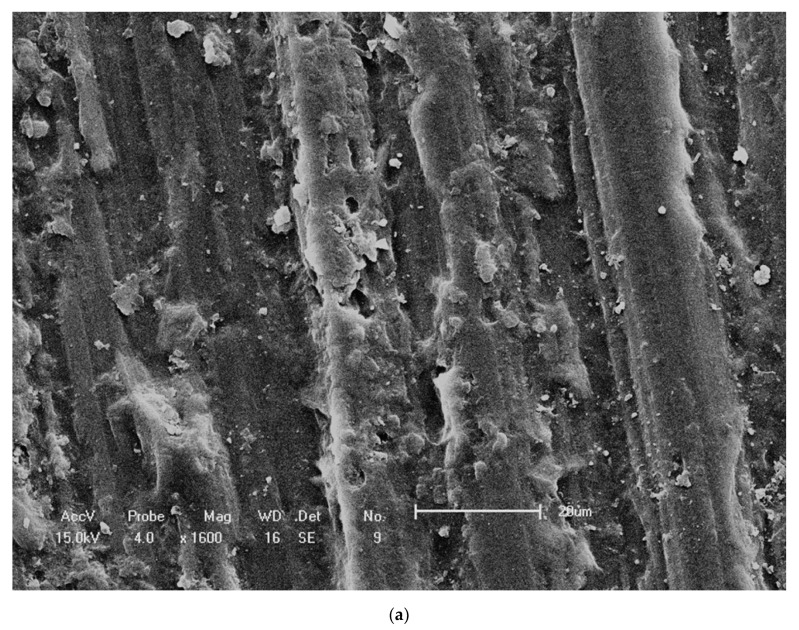

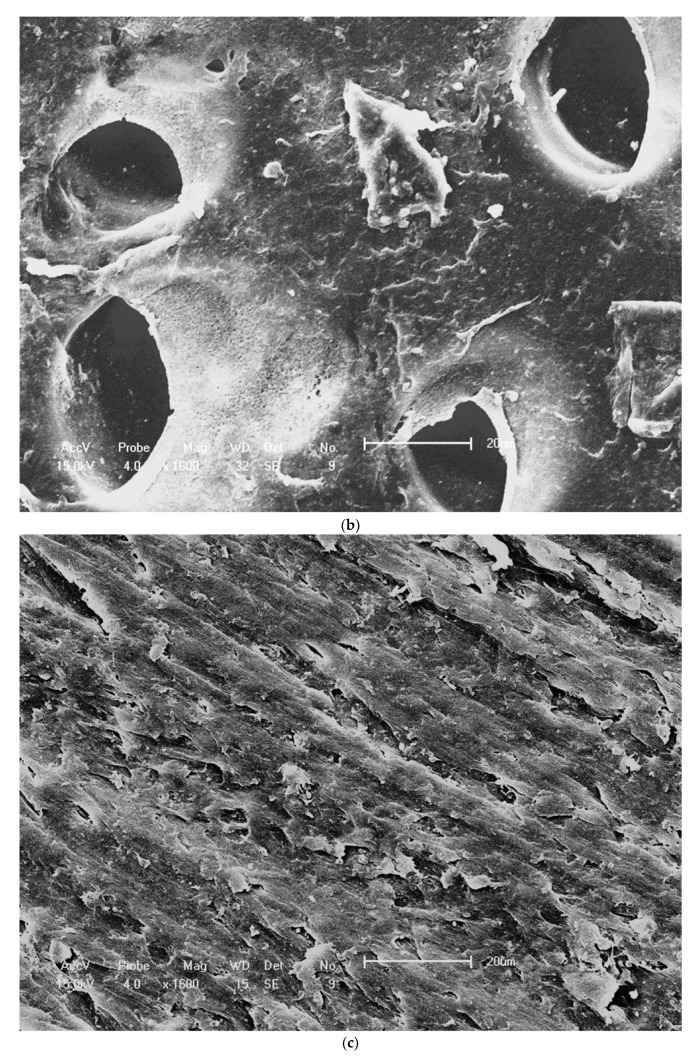

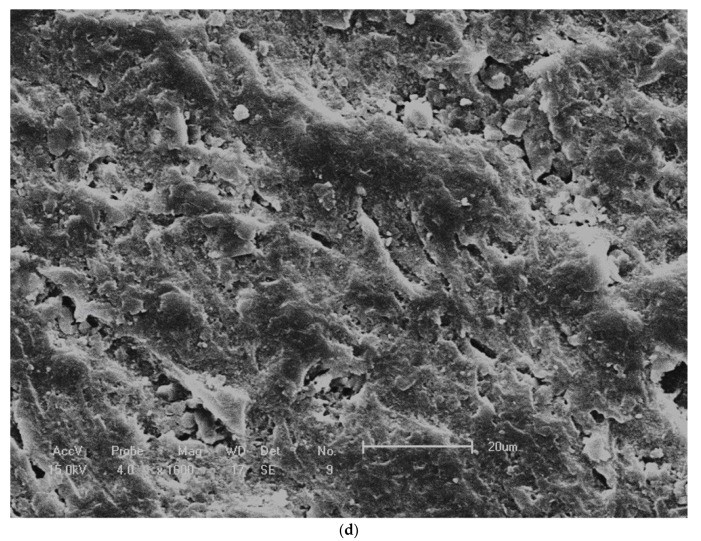
SEM images of PEEK surface with different pretreatments. (**a**) No treatment; (**b**) 98% sulfuric acid; (**c**) 9.5% hydrofluoric acid; (**d**) argon plasma treatment. (Reproduced from [116], with permission from © 2014 Elsevier).

Plasma treatment is an approach to improve the bonding performance of PEEK. After using low-temperature plasma, a pretreated material transforms its non-polar surface into a polar surface and then a dense cross-linked layer or a rough surface is generated. These features all enhance the bonding properties [120]. Several studies focused on the plasma treatment for PEEK. For example, Stawarczyk et al. [34] revealed that a low pressure plasma of helium gas did not improve the adhesive performance of ceramic-filled PEEK to two self-adhesive resin cements. Similar results were found in another study [121]. However, an effective adhesion was obtained by Zhou et al. [116]. They possessed PEEK composites with argon plasma treatment, and the surface of specimens showed some cracks, grooves and deposits, contributing to the enhancement of surface roughness and improvement of the bond strength. The surfaces via different treatments were investigated by scanning electron microscopy (SEM), as shown in Figure 7. The untreated specimens had slight surface scratches (Figure 7a). As shown in Figure 7b, a porous appearance of the tested material was produced after 98% sulfuric acid etching. As shown in Figure 7c, hydrofluoric acid etching presented a limited influence on the surface. As shown in Figure 7d, grooves, cracks and deposits could be observed on its surface via argon plasma treatment.

Bötel et al. [122] evaluated the impact of oxygen and argon/oxygen low-pressure plasma on the shear bond strength (SBS) between different PEEK compounds and three types of veneering cements. Employing the oxygen process gas for a duration of 35 min was shown to be optimal. The effects of the low-pressure plasma compound of argon/oxygen gases between shear bond strength of three types of PEEK (unfilled, ceramic-filled and pigment powder-filled PEEK) and veneering composites were studied by Schwitalla et al. [123]. Meanwhile, through this method in combination with sandblasting, a favorable enhancement in shear bond strength was achieved, especially on unfilled PEEK.

In addition to helium, oxygen and argon, the influence of other types of gases, such as air and nitrogen, on the improvement of PEEK bonding properties has also been studied. For example, Younis et al. [120] showed that plasma treatment of nitrogen gas exerted a positive influence on the SBS of unfilled PEEK and veneering composites. Similar results after nitrogen gas plasma treatment were obtained by Fedel et al. [124]. Therefore, plasma treatment was a suitable surface treatment to improve the bond strength of PEEK materials to dental composites.

### 7.3. Acid Etching

To improve the bond strength of PEEK, attention has been given to acid etching treatments. Studies demonstrated that favorable bonding performance could be acquired after acid etching with 98% sulfuric acid [35,111,116]. PEEK could be etched by a concentrated sulfuric acid, and then highly porous and permeable surfaces to adhesion were formed, thus enhancing the bonding strength [2,111]. Hydrofluoric acid etching had a limited effect on the enhancement of adhesive properties of PEEK [116]. The same unsatisfactory influence on the improvement of bond strength was found in piranha acid etching treatment, which had a less positive effect on the long-term bonding strength between the resin composites and PEEK [31,117].

In addition, the use of sulfuric acid with a high concentration (98%) was risky due to its corrosive nature causing serious damage to mucosa [36,111,116]. Therefore, this shortcoming of 98% sulfuric acid has limited its application in the improvement of PEEK. Although pretreating PEEK with 98% sulfuric acid might be a feasible method, the surface contamination in the dental laboratory and dental office before bonding is a concern and needs to be avoided [111].

### 7.4. Laser Treatment

In order to enhance the poor bond strength of PEEK to luting agents, several approaches of surface treatments, including silica coating, acid etching, air abrasion and plasma treatment, were investigated [30,125]. Recently, as a promising method to improve the bond strength of PEEK, laser irradiation treatment has attracted much attention [52]. However, controversial results were produced. For example, CO_2_ laser treatment did not significantly enhance the SBS of tested PEEK (unfilled PEEK, carbon reinforced PEEK and glass reinforced PEEK) to the resin cement, as shown by Henriques et al. [125]. Similar results that CO_2_ laser treatment had little effect on the inertness and bond strength of the PEEK surface were indicated in another study [126]. Additionally, in that study, the use of Erbium-doped yttrium aluminum garnet (Er: YAG) laser irradiation provided the highest SBS; thus, it could be a feasible approach for improving the adhesive properties of PEEK to the veneers. However, a single pretreatment of Er:YAG laser had no positive and effective influence on improving the adhesion of PEEK to the veneers, as shown by Ates et al. [30]. They also explored the impact of silica coating, airborne-particle abrasion or joint application of Er:YAG laser, revealing that these approaches improved the bonding performance of PEEK to veneers.

In addition to CO_2_ and Er:YAG laser treatments, a neodymium-doped yttrium orthovanadate (Nd:YVO_4_) laser was selected to treat PEEK surface. Uniform laser grooves could be fabricated in the PEEK surface through this type of laser to significantly improve the mechanical interlocking and the bond strength of PEEK to resin-based luting agents [36]. Therefore, laser treatments could serve as a promising method to improve the adhesive properties of PEEK.

### 7.5. PEEK Composites Containing SiO_2_ or TiO_2_

TiO_2_ filler particles had an effect on the bond strength of PEEK, as reported by Lümkemann et al. [127] and Schwitalla et al. [123]. In addition, Rikitoku et al. [128] evaluated the effect of SiO_2_ in PEEK on the bonding strength between PEEK and resin cement. They found that the tensile bonding strength was enhanced with increasing SiO_2_ content in PEEK. PEEK with 40 wt.% SiO_2_ exhibited the highest flexural strength as shown in their results. Therefore, future studies and clinical investigations in the oral environment are needed to evaluate the influence of the type, morphology, particle size, amount and coating of filler particles in PEEK on the long-term durable bonding of PEEK to dental restorations.

Further studies are required to investigate different pretreatment approaches such as etching solutions, various parameters of the air-abrasion treatment, different types of lasers with various laser parameters and different gases in plasma treatments [30]. Importantly, a single pretreatment method of PEEK surface may not be adequate for bonding to resin composites. Instead, a combination of several pretreatments may be a feasible idea.

## 8. Conclusions

PEEK has excellent mechanical properties, wear resistance, stability at high temperatures and good biocompatibility [129]. However, PEEK is bioinert and has a low surface energy which causes difficulties for its potential applications in dentistry [22,42,77,130]. Moreover, PEEK has an opaque and greyish appearance, and thus aesthetic materials such as veneering or resin composites are used to cover it [32,35]. The most recent approaches to modify PEEK were provided in this review, including acid etching, laser treatment, air particle abrasion, plasma treatment and fabricating PEEK composites with special filler particles. The use of 98% sulfuric acid to improve bonding strength is the most effective approach, without inducing any new functional groups. Other strategies also improved the bonding of veneering with PEEK. Further studies in the oral environment and clinical practices are needed in the application of PEEK in dentistry to determine its feasibility and long-term performance.

## Figures and Tables

**Table 1:** Tensile strength and elastic modulus of PEEK, CFR-PEEK and mineralized human tissues. (Reproduced from [38], with permission from © 2016 Elsevier). CFR-PEEK: carbon fiber-reinforced-polyetheretherketone.

Material	Tensile Strength (MPa)	Young’s Modulus (GPa)
PEEK	80	3–4
CFR-PEEK	120	18
Cortical bone	104–121	14
Dentin	104	15
Enamel	47.5	40–83
Titanium	954–976	102–110

**Table 2:** Group distribution, reaction force and maximum principal stress peaks and failure risk for the clasp and the enamel according to the clasp material (elastic modulus - E) and undercut. (Reproduced from [109]. with permission from © 2020 Elsevier).

Clasp Material	Retention (mm)	Reaction Force (N)	Stress Peak (MPa)		Failure Risk	
			**Clasp**	**Enamel**	**Clasp**	**Enamel**
Polyamide (E = 1.44 GPa)	0.25	3.13	17.1	1.4	0.22	0.03
	0.5	6.31	34.2	3.0	0.45	0.07
	0.75	9.47	51.1	4.5	0.68	0.11
Polyoxymethylene (E = 3.15 GPa)	0.25	4.53	45.7	17.2	0.26	0.41
	0.5	8.65	67.8	21.5	0.32	0.51
	0.75	12.31	96.6	35.8	0.54	0.85
Polyetheretherketone-PEEK (E = 3.74 GPa)	0.25	6.45	108.2	26.0	0.33	0.62
	0.5	12.95	134.6	31.1	0.39	0.74
	0.75	18.36	189.9	38.2	0.48	0.91
Gold (E = 91 GPa)	0.25	14.50	160.5	30.2	0.07	0.72
	0.5	28.98	198.2	38.1	0.08	0.90
	0.75	43.41	202.1	44.1	0.10	1.05
Titanium (E = 110 GPa)	0.25	17.81	230.9	32.2	0.07	0.76
	0.5	35.63	241.1	39.8	0.08	0.95
	0.75	53.44	252.2	45.2	0.09	1.07
CoCr (E = 220 GPa)	0.25	21.78	255.8	34.7	0.05	0.82
	0.5	43.57	267.0	41.1	0.06	0.98
	0.75	65.37	297.9	46.4	0.07	1.10

## References

[1] Hallmann L., Mehl A., Sereno N., Hämmerle C.H.F. (2012). The improvement of adhesive properties of PEEK through different pre-treatments. Appl. Surf. Sci..

[2] Schmidlin P.R., Stawarczyk B., Wieland M., Attin T., Hammerle C.H., Fischer J. (2010). Effect of different surface pre-treatments and luting materials on shear bond strength to PEEK. Dent. Mater..

[3] Abdullah M.R., Goharian A., Abdul Kadir M.R., Wahit M.U. (2015). Biomechanical and bioactivity concepts of polyetheretherketone composites for use in orthopedic implants-a review. J. Biomed. Mater. Res. A.

[4] Ma R., Tang T. (2014). Current Strategies to Improve the Bioactivity of PEEK. Int. J. Mol. Sci..

[5] Montero J.F., Tajiri H.A., Barra G.M., Fredel M.C., Benfatti C.A., Magini R.S., Pimenta A.L., Souza J.C. (2017). Biofilm behavior on sulfonated poly(ether-ether-ketone) (sPEEK). Mater. Sci. Eng. C Mater. Biol. Appl..

[6] Zhang J., Tian W., Chen J., Yu J., Zhang J., Chen J. (2019). The application of polyetheretherketone (PEEK) implants in cranioplasty. Brain Res. Bull..

[7] Shimizu T., Fujibayashi S., Yamaguchi S., Otsuki B., Okuzu Y., Matsushita T., Kokubo T., Matsuda S. (2017). In vivo experimental study of anterior cervical fusion using bioactive polyetheretherketone in a canine model. PLoS ONE.

[8] Lee W.T., Koak J.Y., Lim Y.J., Kim S.K., Kwon H.B., Kim M.J. (2012). Stress shielding and fatigue limits of poly-ether-ether-ketone dental implants. J. Biomed. Mater. Res. B Appl. Biomater..

[9] Bataineh K., Al Janaideh M. (2019). Effect of different biocompatible implant materials on the mechanical stability of dental implants under excessive oblique load. Clin. Implant Dent. Relat. Res..

[10] Hahnel S., Scherl C., Rosentritt M. (2018). Interim rehabilitation of occlusal vertical dimension using a double-crown-retained removable dental prosthesis with polyetheretherketone framework. J. Prosthet. Dent..

[11] Zoidis P., Papathanasiou I., Polyzois G. (2016). The Use of a Modified Poly-Ether-Ether-Ketone (PEEK) as an Alternative Framework Material for Removable Dental Prostheses. A Clinical Report. J. Prosthodont..

[12] Zoidis P., Papathanasiou I. (2016). Modified PEEK resin-bonded fixed dental prosthesis as an interim restoration after implant placement. J. Prosthet. Dent..

[13] Uhrenbacher J., Schmidlin P.R., Keul C., Eichberger M., Roos M., Gernet W., Stawarczyk B. (2014). The effect of surface modification on the retention strength of polyetheretherketone crowns adhesively bonded to dentin abutments. J. Prosthet. Dent..

[14] Tannous F., Steiner M., Shahin R., Kern M. (2012). Retentive forces and fatigue resistance of thermoplastic resin clasps. Dent. Mater..

[15] Tada Y., Hayakawa T., Nakamura Y. (2017). Load-Deflection and Friction Properties of PEEK Wires as Alternative Orthodontic Wires. Materials.

[16] Shirakawa N., Iwata T., Miyake S., Otuka T., Koizumi S., Kawata T. (2018). Mechanical properties of orthodontic wires covered with a polyether ether ketone tube. Angle Orthod..

[17] Maekawa M., Kanno Z., Wada T., Hongo T., Doi H., Hanawa T., Ono T., Uo M. (2015). Mechanical properties of orthodontic wires made of super engineering plastic. Dent. Mater. J..

[18] Kurtz S.M., Devine J.N. (2007). PEEK biomaterials in trauma, orthopedic, and spinal implants. Biomaterials.

[19] Rivard C.H., Rhalmi S., Coillard C. (2002). In vivo biocompatibility testing of peek polymer for a spinal implant system: A study in rabbits. J. Biomed. Mater. Res..

[20] Hallab N.J., Bao Q.B., Brown T. (2013). Assessment of epidural versus intradiscal biocompatibility of PEEK implant debris: An in vivo rabbit model. Eur. Spine J..

[21] Nieminen T., Kallela I., Wuolijoki E., Kainulainen H., Hiidenheimo I., Rantala I. (2008). Amorphous and crystalline polyetheretherketone: Mechanical properties and tissue reactions during a 3-year follow-up. J. Biomed. Mater. Res. A.

[22] Wang H., Xu M., Zhang W., Kwok D.T., Jiang J., Wu Z., Chu P.K. (2010). Mechanical and biological characteristics of diamond-like carbon coated poly aryl-ether-ether-ketone. Biomaterials.

[23] Mishra S., Chowdhary R. (2019). PEEK materials as an alternative to titanium in dental implants: A systematic review. Clin. Implant. Dent. Relat. Res..

[24] Park P.J., Lehman R.A. (2020). Optimizing the Spinal Interbody Implant: Current Advances in Material Modification and Surface Treatment Technologies. Curr. Rev. Musculoskelet. Med..

[25] Deng Y., Zhou P., Liu X., Wang L., Xiong X., Tang Z., Wei J., Wei S. (2015). Preparation, characterization, cellular response and in vivo osseointegration of polyetheretherketone/nano-hydroxyapatite/carbon fiber ternary biocomposite. Colloids Surf. B Biointerfaces.

[26] Wachtel A., Zimmermann T., Sutel M., Adali U., Abou-Emara M., Muller W.D., Muhlemann S., Schwitalla A.D. (2019). Bacterial leakage and bending moments of screw-retained, composite-veneered PEEK implant crowns. J. Mech. Behav. Biomed. Mater..

[27] Khalesi R., Abbasi M., Shahidi Z., Tabatabaei M.H., Moradi Z. (2020). Interfacial Fracture Toughness Comparison of Three Indirect Resin Composites to Dentin and Polyether Ether Ketone Polymer. Eur. J. Dent..

[28] Bathala L., Majeti V., Rachuri N., Singh N., Gedela S. (2019). The Role of Polyether Ether Ketone (Peek) in Dentistry—A Review. J. Med. Life.

[29] Stawarczyk B., Taufall S., Roos M., Schmidlin P.R., Lumkemann N. (2018). Bonding of composite resins to PEEK: The influence of adhesive systems and air-abrasion parameters. Clin. Oral Investig..

[30] Ates S.M., Caglar I., Yesil Duymus Z. (2018). The effect of different surface pretreatments on the bond strength of veneering resin to polyetheretherketone. J. Adhes. Sci. Technol..

[31] Keul C., Liebermann A., Schmidlin P.R., Roos M., Sener B., Stawarczyk B. (2014). Influence of PEEK surface modification on surface properties and bond strength to veneering resin composites. J. Adhes. Dent..

[32] Stawarczyk B., Keul C., Beuer F., Roos M., Schmidlin P.R. (2013). Tensile bond strength of veneering resins to PEEK: Impact of different adhesives. Dent. Mater. J..

[33] Panayotov I.V., Orti V., Cuisinier F., Yachouh J. (2016). Polyetheretherketone (PEEK) for medical applications. J. Mater. Sci. Mater. Med..

[34] Stawarczyk B., Bahr N., Beuer F., Wimmer T., Eichberger M., Gernet W., Jahn D., Schmidlin P.R. (2014). Influence of plasma pretreatment on shear bond strength of self-adhesive resin cements to polyetheretherketone. Clin. Oral Investig..

[35] Silthampitag P., Chaijareenont P., Tattakorn K., Banjongprasert C., Takahashi H., Arksornnukit M. (2016). Effect of surface pretreatments on resin composite bonding to PEEK. Dent. Mater. J..

[36] Tsuka H., Morita K., Kato K., Kimura H., Abekura H., Hirata I., Kato K., Tsuga K. (2019). Effect of laser groove treatment on shear bond strength of resin-based luting agent to polyetheretherketone (PEEK). J. Prosthodont. Res..

[37] Kaleli N., Sarac D., Kulunk S., Ozturk O. (2018). Effect of different restorative crown and customized abutment materials on stress distribution in single implants and peripheral bone: A three-dimensional finite element analysis study. J. Prosthet. Dent..

[38] Najeeb S., Zafar M.S., Khurshid Z., Siddiqui F. (2016). Applications of polyetheretherketone (PEEK) in oral implantology and prosthodontics. J. Prosthodont. Res..

[39] Gan K., Liu H., Jiang L., Liu X., Song X., Niu D., Chen T., Liu C. (2016). Bioactivity and antibacterial effect of nitrogen plasma immersion ion implantation on polyetheretherketone. Dent. Mater..

[40] Knaus J., Schaffarczyk D., Colfen H. (2020). On the Future Design of Bio-Inspired Polyetheretherketone Dental Implants. Macromol. Biosci..

[41] Schwitalla A., Müller W.-D. (2013). PEEK Dental Implants: A Review of the Literature. J. Oral Implantol..

[42] Wang W., Luo C.J., Huang J., Edirisinghe M. (2019). PEEK surface modification by fast ambient-temperature sulfonation for bone implant applications. J. R. Soc. Interface.

[43] Alexakou E., Damanaki M., Zoidis P., Bakiri E., Mouzis N., Smidt G., Kourtis S. (2019). PEEK High Performance Polymers: A Review of Properties and Clinical Applications in Prosthodontics and Restorative Dentistry. Eur. J. Prosthodont. Restor. Dent..

[44] Werner P., Altstädt V., Jaskulka R., Jacobs O., Sandler J.K.W., Shaffer M.S.P., Windle A.H. (2004). Tribological behaviour of carbon-nanofibre-reinforced poly(ether ether ketone). Wear.

[45] Ouyang L., Zhao Y., Jin G., Lu T., Li J., Qiao Y., Ning C., Zhang X., Chu P.K., Liu X. (2016). Influence of sulfur content on bone formation and antibacterial ability of sulfonated PEEK. Biomaterials.

[46] Buck E., Li H., Cerruti M. (2020). Surface Modification Strategies to Improve the Osseointegration of Poly(etheretherketone) and Its Composites. Macromol. Biosci..

[47] Rahmitasari F., Ishida Y., Kurahashi K., Matsuda T., Watanabe M., Ichikawa T. (2017). PEEK with Reinforced Materials and Modifications for Dental Implant Applications. Dent. J..

[48] Wang L., He S., Wu X., Liang S., Mu Z., Wei J., Deng F., Deng Y., Wei S. (2014). Polyetheretherketone/nano-fluorohydroxyapatite composite with antimicrobial activity and osseointegration properties. Biomaterials.

[49] Rabiei A., Sandukas S. (2013). Processing and evaluation of bioactive coatings on polymeric implants. J. Biomed. Mater. Res. Part A.

[50] Walsh W.R., Bertollo N., Christou C., Schaffner D., Mobbs R.J. (2015). Plasma-sprayed titanium coating to polyetheretherketone improves the bone-implant interface. Spine J..

[51] Benli M., Eker Gumus B., Kahraman Y., Huck O., Ozcan M. (2020). Surface characterization and bonding properties of milled polyetheretherketone dental posts. Odontology.

[52] Guo L., Smeets R., Kluwe L., Hartjen P., Barbeck M., Cacaci C., Gosau M., Henningsen A. (2019). Cytocompatibility of Titanium, Zirconia and Modified PEEK after Surface Treatment Using UV Light or Non-Thermal Plasma. Int. J. Mol. Sci..

[53] Katzer A., Marquardt H., Westendorf J., Wening J.V., von Foerster G. (2002). Polyetheretherketone--cytotoxicity and mutagenicity in vitro. Biomaterials.

[54] Wenz L.M., Merritt K., Brown S.A., Moet A., Steffee A.D. (1990). In vitro biocompatibility of polyetheretherketone and polysulfone composites. J. Biomed. Mater. Res..

[55] Morrison C., Macnair R., Macdonald C., Wykman A., Goldie I., Grant M.H. (1995). In vitro biocompatibility testing of polymers for orthopaedic implants using cultured fibroblasts and osteoblasts. Biomaterials.

[56] Hepdarcan S.S., Yilmaz R.B.N., Nalbantgil D. (2016). Which Orthodontic Wire and Working Sequence Should be Preferred for Alignment Phase? A Review. Turk. J. Orthod..

[57] Ierardo G., Luzzi V., Lesti M., Vozza I., Brugnoletti O., Polimeni A., Bossu M. (2017). Peek polymer in orthodontics: A pilot study on children. J. Clin. Exp. Dent..

[58] Rokaya D., Srimaneepong V., Sapkota J., Qin J., Siraleartmukul K., Siriwongrungson V. (2018). Polymeric materials and films in dentistry: An overview. J. Adv. Res..

[59] da Silva D.L., Mattos C.T., Simao R.A., de Oliveira Ruellas A.C. (2013). Coating stability and surface characteristics of esthetic orthodontic coated archwires. Angle Orthod..

[60] Elawadly T., Radi I.A.W., El Khadem A., Osman R.B. (2017). Can PEEK Be an Implant Material? Evaluation of Surface Topography and Wettability of Filled Versus Unfilled PEEK with Different Surface Roughness. J. Oral Implantol..

[61] Tekin S., Deger Y., Demirci F. (2019). Evaluation of the use of PEEK material in implant-supported fixed restorations by finite element analysis. Niger. J. Clin. Pract..

[62] Toth J.M., Wang M., Estes B.T., Scifert J.L., Seim H.B., Turner A.S. (2006). Polyetheretherketone as a biomaterial for spinal applications. Biomaterials.

[63] Wang S., Yang Y., Li Y., Shi J., Zhou J., Zhang L., Deng Y., Yang W. (2019). Strontium/adiponectin co-decoration modulates the osteogenic activity of nano-morphologic polyetheretherketone implant. Colloids Surf. B: Biointerfaces.

[64] Steinberg E.L., Rath E., Shlaifer A., Chechik O., Maman E., Salai M. (2013). Carbon fiber reinforced PEEK Optima--a composite material biomechanical properties and wear/debris characteristics of CF-PEEK composites for orthopedic trauma implants. J. Mech. Behav. Biomed. Mater..

[65] Sumner D.R. (2015). Long-term implant fixation and stress-shielding in total hip replacement. J. Biomech..

[66] Korabi R., Shemtov-Yona K., Rittel D. (2017). On stress/strain shielding and the material stiffness paradigm for dental implants. Clin. Implantol. Dent. Relat. Res..

[67] Carpenter R.D., Klosterhoff B.S., Torstrick F.B., Foley K.T., Burkus J.K., Lee C.S.D., Gall K., Guldberg R.E., Safranski D.L. (2018). Effect of porous orthopaedic implant material and structure on load sharing with simulated bone ingrowth: A finite element analysis comparing titanium and PEEK. J. Mech. Behav. Biomed. Mater..

[68] Anguiano-Sanchez J., Martinez-Romero O., Siller H.R., Diaz-Elizondo J.A., Flores-Villalba E., Rodriguez C.A. (2016). Influence of PEEK Coating on Hip Implant Stress Shielding: A Finite Element Analysis. Comput. Math. Methods Med..

[69] Sarot J.R., Contar C.M., Cruz A.C., de Souza Magini R. (2010). Evaluation of the stress distribution in CFR-PEEK dental implants by the three-dimensional finite element method. J. Mater. Sci. Mater. Med..

[70] Olivares-Navarrete R., Gittens R.A., Schneider J.M., Hyzy S.L., Haithcock D.A., Ullrich P.F., Schwartz Z., Boyan B.D. (2012). Osteoblasts exhibit a more differentiated phenotype and increased bone morphogenetic protein production on titanium alloy substrates than on poly-ether-ether-ketone. Spine J..

[71] Sunarso, Tsuchiya A., Toita R., Tsuru K., Ishikawa K. (2019). Enhanced Osseointegration Capability of Poly(ether ether ketone) via Combined Phosphate and Calcium Surface-Functionalization. Int. J. Mol. Sci..

[72] Cheng B.C., Koduri S., Wing C.A., Woolery N., Cook D.J., Spiro R.C. (2018). Porous titanium-coated polyetheretherketone implants exhibit an improved bone-implant interface: An in vitro and in vivo biochemical, biomechanical, and histological study. Med. Devices.

[73] Wan T., Jiao Z., Guo M., Wang Z., Wan Y., Lin K., Liu Q., Zhang P. (2020). Gaseous sulfur trioxide induced controllable sulfonation promoting biomineralization and osseointegration of polyetheretherketone implants. Bioact. Mater..

[74] Zhao Y., Wong H.M., Wang W., Li P., Xu Z., Chong E.Y., Yan C.H., Yeung K.W., Chu P.K. (2013). Cytocompatibility, osseointegration, and bioactivity of three-dimensional porous and nanostructured network on polyetheretherketone. Biomaterials.

[75] Li Y., Wang J., He D., GuoxiongZhu, Wu G., Chen L. (2019). Surface sulfonation and nitrification enhance the biological activity and osteogenesis of polyetheretherketone by forming an irregular nano-porous monolayer. J. Mater. Sci. Mater. Med..

[76] Wang H., Lu T., Meng F., Zhu H., Liu X. (2014). Enhanced osteoblast responses to poly ether ether ketone surface modified by water plasma immersion ion implantation. Colloids Surf. B Biointerfaces.

[77] Zheng Y., Xiong C., Zhang S., Li X., Zhang L. (2015). Bone-like apatite coating on functionalized poly(etheretherketone) surface via tailored silanization layers technique. Mater. Sci. Eng. C Mater. Biol. Appl..

[78] Yu X., Ibrahim M., Liu Z., Yang H., Tan L., Yang K. (2018). Biofunctional Mg coating on PEEK for improving bioactivity. Bioact. Mater..

[79] Najeeb S., Khurshid Z., Matinlinna J.P., Siddiqui F., Nassani M.Z., Baroudi K. (2015). Nanomodified Peek Dental Implants: Bioactive Composites and Surface Modification-A Review. Int. J. Dent..

[80] Wu X., Liu X., Wei J., Ma J., Deng F., Wei S. (2012). Nano-TiO2/PEEK bioactive composite as a bone substitute material: In vitro and in vivo studies. Int. J. Nanomed..

[81] Xu A., Liu X., Gao X., Deng F., Deng Y., Wei S. (2015). Enhancement of osteogenesis on micro/nano-topographical carbon fiber-reinforced polyetheretherketone-nanohydroxyapatite biocomposite. Mater. Sci. Eng. C Mater. Biol. Appl..

[82] Almasi D., Lau W.J., Rasaee S., Sharifi R., Mozaffari H.R. (2020). Fabrication of a novel hydroxyapatite/polyether ether ketone surface nanocomposite via friction stir processing for orthopedic and dental applications. Prog. Biomater..

[83] Brum R.S., Labes L.G., Volpato C.A.M., Benfatti C.A.M., Pimenta A.L. (2020). Strategies to Reduce Biofilm Formation in PEEK Materials Applied to Implant Dentistry-A Comprehensive Review. Antibiotics.

[84] Garcia D., Mayfield C.K., Leong J., Deckey D.G., Zega A., Glasser J., Daniels A.H., Eberson C., Green A., Born C. (2020). Early adherence and biofilm formation of Cutibacterium acnes (formerly Propionibacterium acnes) on spinal implant materials. Spine J..

[85] Hahnel S., Wieser A., Lang R., Rosentritt M. (2015). Biofilm formation on the surface of modern implant abutment materials. Clin. Oral Implantol. Res..

[86] Santing H.J., Meijer H.J., Raghoebar G.M., Ozcan M. (2012). Fracture strength and failure mode of maxillary implant-supported provisional single crowns: A comparison of composite resin crowns fabricated directly over PEEK abutments and solid titanium abutments. Clin. Implantol. Dent. Relat. Res..

[87] Koutouzis T., Richardson J., Lundgren T. (2011). Comparative soft and hard tissue responses to titanium and polymer healing abutments. J. Oral Implantol..

[88] Mate Sanchez de Val J.E., Gomez-Moreno G., Perez-Albacete Martinez C., Ramirez-Fernandez M.P., Granero-Marin J.M., Gehrke S.A., Calvo-Guirado J.L. (2016). Peri-implant tissue behavior around non-titanium material: Experimental study in dogs. Ann. Anat..

[89] Pan Y., Tam J.M.Y., Tsoi J.K.H., Lam W.Y.H., Pow E.H.N. (2020). Reproducibility of laboratory scanning of multiple implants in complete edentulous arch: Effect of scan bodies. J. Dent..

[90] Mizumoto R.M., Yilmaz B. (2018). Intraoral scan bodies in implant dentistry: A systematic review. J. Prosthet. Dent..

[91] Arcuri L., Pozzi A., Lio F., Rompen E., Zechner W., Nardi A. (2020). Influence of implant scanbody material, position and operator on the accuracy of digital impression for complete-arch: A randomized in vitro trial. J. Prosthodont. Res..

[92] Nazari V., Ghodsi S., Alikhasi M., Sahebi M., Shamshiri A.R. (2016). Fracture Strength of Three-Unit Implant Supported Fixed Partial Dentures with Excessive Crown Height Fabricated from Different Materials. J. Dent..

[93] Elsayed A., Farrag G., Chaar M.S., Abdelnabi N., Kern M. (2019). Influence of Different CAD/CAM Crown Materials on the Fracture of Custom-Made Titanium and Zirconia Implant Abutments After Artificial Aging. Int. J. Prosthodont..

[94] Neumann E.A., Villar C.C., Franca F.M. (2014). Fracture resistance of abutment screws made of titanium, polyetheretherketone, and carbon fiber-reinforced polyetheretherketone. Braz. Oral Res..

[95] Stimmelmayr M., Lang A., Beuer F., Mansour S., Erdelt K., Krennmair G., Guth J.F. (2020). Mechanical stability of all-ceramic abutments retained with three different screw materials in two-piece zirconia implants-an in vitro study. Clin. Oral Investig..

[96] Stawarczyk B., Schmid P., Roos M., Eichberger M., Schmidlin P.R. (2016). Spectrophotometric Evaluation of Polyetheretherketone (PEEK) as a Core Material and a Comparison with Gold Standard Core Materials. Materials.

[97] Schubert O., Reitmaier J., Schweiger J., Erdelt K., Guth J.F. (2019). Retentive force of PEEK secondary crowns on zirconia primary crowns over time. Clin. Oral Investig..

[98] Stock V., Schmidlin P.R., Merk S., Wagner C., Roos M., Eichberger M., Stawarczyk B. (2016). PEEK Primary Crowns with Cobalt-Chromium, Zirconia and Galvanic Secondary Crowns with Different Tapers-A Comparison of Retention Forces. Materials.

[99] Merk S., Wagner C., Stock V., Eichberger M., Schmidlin P.R., Roos M., Stawarczyk B. (2016). Suitability of Secondary PEEK Telescopic Crowns on Zirconia Primary Crowns: The Influence of Fabrication Method and Taper. Materials.

[100] Stawarczyk B., Eichberger M., Uhrenbacher J., Wimmer T., Edelhoff D., Schmidlin P.R. (2015). Three-unit reinforced polyetheretherketone composite FDPs: Influence of fabrication method on load-bearing capacity and failure types. Dent. Mater. J..

[101] Saeed F., Muhammad N., Khan A.S., Sharif F., Rahim A., Ahmad P., Irfan M. (2020). Prosthodontics dental materials: From conventional to unconventional. Mater. Sci. Eng. C Mater. Biol. Appl..

[102] Hu F., Pei Z., Wen Y. (2019). Using Intraoral Scanning Technology for Three-Dimensional Printing of Kennedy Class I Removable Partial Denture Metal Framework: A Clinical Report. J. Prosthodont..

[103] Pagano S., Moretti M., Marsili R., Ricci A., Barraco G., Cianetti S. (2019). Evaluation of the Accuracy of Four Digital Methods by Linear and Volumetric Analysis of Dental Impressions. Materials.

[104] Harb I.E., Abdel-Khalek E.A., Hegazy S.A. (2019). CAD/CAM Constructed Poly(etheretherketone) (PEEK) Framework of Kennedy Class I Removable Partial Denture: A Clinical Report. J. Prosthodont..

[105] Pacurar M., Bechir E.S., Suciu M., Bechir A., Biris C.I., Mola F.C., Gioga C., Dascalu I.T., Ormenisan A. (2016). The Benefits of Polyether-Ether-Ketone Polymers in Partial Edentulous Patients. Mater. Plast..

[106] Negm E.E., Aboutaleb F.A., Alam-Eldein A.M. (2019). Virtual Evaluation of the Accuracy of Fit and Trueness in Maxillary Poly(etheretherketone) Removable Partial Denture Frameworks Fabricated by Direct and Indirect CAD/CAM Techniques. J. Prosthodont..

[107] Sadek S.A. (2019). Comparative Study Clarifying the Usage of PEEK as Suitable Material to Be Used as Partial Denture Attachment and Framework. Open Access Maced. J. Med. Sci..

[108] Ali Z., Baker S., Sereno N., Martin N. (2020). A Pilot Randomized Controlled Crossover Trial Comparing Early OHRQoL Outcomes of Cobalt-Chromium Versus PEEK Removable Partial Denture Frameworks. Int. J. Prosthodont..

[109] Tribst J.P.M., Dal Piva A.M.O., Borges A.L.S., Araujo R.M., da Silva J.M.F., Bottino M.A., Kleverlaan C.J., de Jager N. (2020). Effect of different materials and undercut on the removal force and stress distribution in circumferential clasps during direct retainer action in removable partial dentures. Dent. Mater..

[110] Peng T.Y., Ogawa Y., Akebono H., Iwaguro S., Sugeta A., Shimoe S. (2020). Finite-element analysis and optimization of the mechanical properties of polyetheretherketone (PEEK) clasps for removable partial dentures. J. Prosthodont. Res..

[111] Stawarczyk B., Beuer F., Wimmer T., Jahn D., Sener B., Roos M., Schmidlin P.R. (2013). Polyetheretherketone-a suitable material for fixed dental prostheses?. J. Biomed. Mater. Res. Part B Appl. Biomater..

[112] Alt V., Hannig M., Wostmann B., Balkenhol M. (2011). Fracture strength of temporary fixed partial dentures: CAD/CAM versus directly fabricated restorations. Dent. Mater..

[113] Cekic-Nagas I., Egilmez F., Ergun G., Vallittu P.K., Lassila L.V.J. (2018). Load-bearing capacity of novel resin-based fixed dental prosthesis materials. Dent. Mater. J..

[114] Goncu Basaran E., Ayna E., Vallittu P.K., Lassila L.V. (2011). Load-bearing capacity of handmade and computer-aided design—Computer-aided manufacturing-fabricated three-unit fixed dental prostheses of particulate filler composite. Acta Odontol. Scand..

[115] Sinha N., Gupta N., Reddy K.M., Shastry Y.M. (2017). Versatility of PEEK as a fixed partial denture framework. J. Indian Prosthodont. Soc..

[116] Zhou L., Qian Y., Zhu Y., Liu H., Gan K., Guo J. (2014). The effect of different surface treatments on the bond strength of PEEK composite materials. Dent. Mater..

[117] Stawarczyk B., Jordan P., Schmidlin P.R., Roos M., Eichberger M., Gernet W., Keul C. (2014). PEEK surface treatment effects on tensile bond strength to veneering resins. J. Prosthet. Dent..

[118] Culhaoglu A.K., Ozkir S.E., Sahin V., Yilmaz B., Kilicarslan M.A. (2020). Effect of Various Treatment Modalities on Surface Characteristics and Shear Bond Strengths of Polyetheretherketone-Based Core Materials. J. Prosthodont..

[119] Fokas G., Guo C.Y., Tsoi J.K.H. (2019). The effects of surface treatments on tensile bond strength of polyether-ketone-ketone (PEKK) to veneering resin. J. Mech. Behav. Biomed. Mater..

[120] Younis M., Unkovskiy A., ElAyouti A., Geis-Gerstorfer J., Spintzyk S. (2019). The Effect of Various Plasma Gases on the Shear Bond Strength between Unfilled Polyetheretherketone (PEEK) and Veneering Composite Following Artificial Aging. Materials.

[121] Schmidlin P.R., Eichberger M., Stawarczyk B. (2016). Glycine: A potential coupling agent to bond to helium plasma treated PEEK?. Dent. Mater..

[122] Botel F., Zimmermann T., Sutel M., Muller W.D., Schwitalla A.D. (2018). Influence of different low-pressure plasma process parameters on shear bond strength between veneering composites and PEEK materials. Dent. Mater..

[123] Schwitalla A.D., Botel F., Zimmermann T., Sutel M., Muller W.D. (2017). The impact of argon/oxygen low-pressure plasma on shear bond strength between a veneering composite and different PEEK materials. Dent. Mater..

[124] Fedel M., Micheli V., Thaler M., Awaja F. (2020). Effect of nitrogen plasma treatment on the crystallinity and self-bonding of polyetheretherketone (PEEK) for biomedical applications. Polym. Adv. Technol..

[125] Henriques B., Fabris D., Mesquita-Guimaraes J., Sousa A.C., Hammes N., Souza J.C.M., Silva F.S., Fredel M.C. (2018). Influence of laser structuring of PEEK, PEEK-GF30 and PEEK-CF30 surfaces on the shear bond strength to a resin cement. J. Mech. Behav. Biomed. Mater..

[126] Jahandideh Y., Falahchai M., Pourkhalili H. (2020). Effect of Surface Treatment With Er:YAG and CO2 Lasers on Shear Bond Strength of Polyether Ether Ketone to Composite Resin Veneers. J. Lasers Med. Sci..

[127] Lumkemann N., Eichberger M., Stawarczyk B. (2017). Bonding to Different PEEK Compositions: The Impact of Dental Light Curing Units. Materials.

[128] Rikitoku S., Otake S., Nozaki K., Yoshida K., Miura H. (2019). Influence of SiO2 content of polyetheretherketone (PEEK) on flexural properties and tensile bond strength to resin cement. Dent. Mater. J..

[129] Honigmann P., Sharma N., Okolo B., Popp U., Msallem B., Thieringer F.M. (2018). Patient-Specific Surgical Implants Made of 3D Printed PEEK: Material, Technology, and Scope of Surgical Application. BioMed Res. Int..

[130] Yu W., Zhang H., Lan A., Yang S., Zhang J., Wang H., Zhou Z., Zhou Y., Zhao J., Jiang Z. (2020). Enhanced bioactivity and osteogenic property of carbon fiber reinforced polyetheretherketone composites modified with amino groups. Colloids Surf. B Biointerfaces.

